# Long-term sequelae after viral meningitis and meningoencephalitis are frequent, even in mildly affected patients, a prospective observational study

**DOI:** 10.3389/fneur.2024.1411860

**Published:** 2024-07-17

**Authors:** Janine Schwitter, Mattia Branca, Antonela Bicvic, Lena S. Abbuehl, Franziska Suter-Riniker, Stephen L. Leib, Anelia Dietmann

**Affiliations:** ^1^Department of Neurology, Inselspital, Bern University Hospital, and University of Bern, Bern, Switzerland; ^2^CTU Bern, University of Bern, Bern, Switzerland; ^3^Institute for Infectious Diseases, University of Bern, Bern, Switzerland

**Keywords:** viral encephalitis, tick-borne encephalitis, meningoencephalitis, outcome, long-term sequalae, meningitis

## Abstract

**Introduction:**

An increasing number of studies demonstrate that viral meningitis and meningoencephalitis, even those with a mild course of meningitis, can result in residual sequelae.

**Methods:**

We aimed to investigate the long-term outcome in both viral meningitis and meningoencephalitis/encephalitis patients and impact of long-term sequelae on patients’ social and professional daily lives in a prospective observational study with a follow-up period of 20 months.

**Results:**

A total of 50 patients (12% encephalitis, 58% meningoencephalitis and 30% meningitis) and 21 control persons participated in the study. The most common cause was the tick-borne encephalitis (TBE) virus. The most important persistent signs and symptoms after 2 years were subjective cognitive impairment (36%), fatigue and/or excessive daytime sleepiness (31%), disturbed nighttime sleep (31%) and headaches (13%), as well as feeling more rapidly exhausted after cognitive effort (53%). Independent of disease severity in the acute phase, almost one third of patients still reported mildly impaired social and/or professional life due to the long-term sequelae, with scores in the health status assessment still significantly lower compared to healthy controls.

**Discussion:**

Regardless of the severity of the acute illness and despite constant improvement within 2 years, 67% of patients still had persistent signs and symptoms, but these were only relevant to everyday social or professional life in about 30% of these patients.

## 1 Introduction

Encephalitis and meningoencephalitis are medical conditions characterized by inflammation of the brain parenchyma, with or without involvement of meningeal structures, while meningitis, by definition, affects only the meninges. Clinically, the boundaries between encephalitis, meningoencephalitis and meningitis are often fluid and not easy to establish. Besides bacteria and autoimmune etiology, viruses are the main causes of encephalitis and meningitis, although between 30 and 60% of cases have no known cause (1–5). Depending on the region of the world, HSV is the most common pathogen causing viral encephalitis in western industrialized nations (1, 3, 5, 6) and enteroviruses are most frequently found in cases of meningitis (4, 5). However, the frequency with which viruses causing encephalitis or meningitis are detected depends on the geographical location; for instance, in Switzerland, the tick-borne encephalitis (TBE) virus is the most frequently encountered pathogen causing meningitis, meningoencephalitis and sometimes meningomyeloradiculitis (5).

Although large multicenter studies often report a poor long-term outcome in encephalitis patients, with less than half of them experiencing full or good recovery (1, 7, 8), viral meningitis is considered to have a benign course leading to full recovery (9–11). In TBE, long-term sequelae have been described in up to 33% of patients (12, 13). Commonly described long-term sequelae are headache, cognitive impairment, such as memory and attention disturbances, as well as balance/coordination dysfunction, and mental health problems (4, 5, 13–15). Importantly, an increasing number of studies demonstrate that viral meningitis, even if it has a mild course, can result in residual sequelae (15–18). Often these go undetected in clinical routine, since follow-up consultations may be not being offered to patients with mild courses.

The working hypothesis of our study was that not only severe encephalitis but also mild forms of viral meningitis can cause permanent long-term sequelae. Therefore, the aim of our prospective study was to investigate the influence of the severity of the disease during the acute phase on the long-term outcome. Secondly, we wanted to investigate the impact of the persisting long-term sequelae on the patients’ social and professional daily life.

## 2 Materials and methods

A prospective, single-center observational cohort study was designed and carried out.

### 2.1 Participants, inclusion criteria and definitions

Ethical approval was given by the local Ethics Committee (Kantonale Ethik Kommission Bern 2019–00300) and research governance approval by the University Hospital Inselspital, Bern, Switzerland. All methods were performed in accordance with the relevant guidelines and regulations and were in accordance with the Declaration of Helsinki (19). From June 2019 until December 2021, an automated screening of the digital clinical patient information system was performed twice a week by the Insel Data Science Center for new patient admissions. The Insel Data Science Center (IDSC) is a cross-divisional organizational unit of Insel Gruppe AG for the collection, provision and use of digital data from the Insel Gruppe Ag. An IDSC employee carried out queries in the hospital’s internal digital medical record system to find patients who had recently been admitted to Inselspital. The following search terms were used: “encephalitis,” “meningoencephalitis,” “meningitis,” “tick-borne encephalitis,” “herpes simplex virus.” Only patients who could be found under these search terms were sent to a member of our study group (AD) with identifiers for screening. These patients with these diagnoses were screened for inclusion and exclusion criteria. Patients eligible for study participation were contacted by a member of the study team and enrolled in the study after signing informed consent.

To be eligible, participants needed to be at least 18 years of age, require hospitalization and provide written informed consent. Meningitis was defined corresponding to published criteria (16, 20): fever ≥38°C and/or headache and/or meningism and the presence of signs and symptoms for more than 24 h, without any alternative diagnosis. At least one of the following criteria had to be met: Infectious constellation in the cerebrospinal fluid (CSF; cell count ≥5 leukocytes/ml); brain imaging suggesting meningitis of recent onset; detection of an appropriate pathogen in either blood or CSF, by polymerase chain reaction (PCR), throat or rectal swab, or serology. All patients who were included in the study after the start of the SARS-CoV-2 pandemic were tested for COVID-19 by PCR as part of the routine clinical examination.

The inclusion criteria for meningoencephalitis and encephalitis were set according to published case definitions and international guidelines (21, 22): altered mental status (including altered consciousness, lethargy, irritability, or change in personality) and presence of signs and symptoms for more than 24 h without any alternative diagnosis. Two or more of the following signs had to be present: fever (≥38°C); seizures or focal neurological signs; CSF pleocytosis (defined as ≥5 leukocytes/ml); electroencephalogram (EEG) suggesting encephalitis; and neuroimaging suggestive of encephalitis; detection of an appropriate pathogen from either blood or CSF, by PCR, throat or rectal swab, or serology.

The exclusion criteria were: purulent bacterial meningitis; autoimmune encephalitis, acute disseminated encephalomyelitis or other chronic inflammatory or infectious central nervous system (CNS) diseases (e.g., brain abscess, CNS malignancy, CNS vasculitis or cerebral venous thrombosis, if not associated with encephalitis).

We also included a group of healthy volunteers who met the following inclusion criteria: Age ≥ 18 years (matched for age and sex); written informed consent; no documented neurological disease/disorder requiring regular treatment; no subjectively reported excessive daytime sleepiness (EDS), fatigue or disturbed nighttime sleep.

### 2.2 Study procedure

Epidemiological, clinical, radiological (MRI/CT), electrophysiological (EEG) and laboratory results (serum/CSF) of study participants were transferred into a REDCap Database (23, 24) from the clinical record and were completed during a personal interview and examination. Furthermore, during the hospital stay in the acute phase and in a follow-up visit in the year after hospital discharge, patients underwent a detailed neurological examination and neurocognitive testing [Addenbrook’s cognitive examination (ACE) III]. Patients also filled out a set of questionnaires [Epworth Sleepiness Scale (ESS), Fatigue Severity Score (FSS), Insomnia Severity Index (ISI), Beck Depression Inventory II (BDI II), general life quality questionnaires (sf-36 and EQ-5D-5L); detailed explanations of the scores/questionnaires are provided in the Supplementary data] and wore an actigraph for 7 consecutive days. Actigraphy is a methodology based on small watch-like portable devices worn around the non-dominant wrist that collect movement information for extended periods. The aim is to monitor sleep–wake patterns and rest-activity cycles.

Another follow-up consultation 2–3 years after hospital discharge was performed via a telephone interview, which included questionnaires.

### 2.3 Severity grading

Disease severity was classified into 3 groups, mild, moderate and severe, according to published case definitions (13, 14, 25, 26). Mild was defined as mainly meningeal symptoms; moderate as monofocal symptoms of the CNS and/or moderate diffuse brain dysfunction; and severe as multifocal CNS symptoms and/or severe diffuse brain dysfunction with altered consciousness.

### 2.4 Statistical analysis

The statistical analysis was performed using the Stata software, version 17.0. Descriptive statistics were obtained using frequencies and percentages for categorical variables and mean with standard deviation or median with interquartile range for continuous variables. To compare the different groups and follow-up time points, ANOVA and Kruskal-Wallis tests were applied for continuous variables, while Fisher’s exact test was applied for categorial variables. To estimate the influence and correlation of different factors on the outcomes a logistic regression on the complete cases was applied and the corresponding odds ratios (OR) with 95% confidence interval (CI) were derived to describe the relationship between the outcomes and the predictors. Logistic models were used with social/professional limitations or with modified Rankin scale mRS dichotomized as outcome. The OR represent the odds of limitations or mRS > 0 of the presence of the condition or the increase of a unit (continuous variable).

## 3 Results

**Figure 1:**
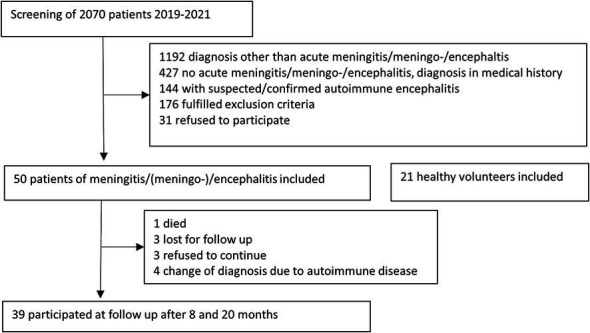
Screening and inclusion of study participants.

Out of 2070 patients screened, 50 were eligible for participation and were enrolled in the study (Figure 1). Of these patients, 63% were male and the median age was 43 years (IQR 32, 62). More than half had at least one underlying illness such as diabetes (*n* = 3), cardiovascular disease (*n* = 3), heart failure (*n* = 3), renal insufficiency (*n* = 3), lung disease (*n* = 1), systemic disease (*n* = 2), tumor disease in remission (*n* = 8), neurological disorder including headache (*n* = 13), immunodeficiency (*n* = 5), history of encephalitis or meningitis (*n* = 1), wake–sleep disorder (*n* = 14). In the control group (*n* = 21), 57% were men and the median age was 42 years (IQR 27, 62). Three participants in the control group had pre-existing conditions (cardiovascular disease *n* = 3, diabetes *n* = 1). TBE vaccination status was complete in only 4 patients, and partial in another 3, none of the TBE patients had received vaccination.

The most common cause, affecting almost a third of the patients (32%, *n* = 16), was TBE virus, diagnosed by positive IgM serology. Other causes were Varicella Zoster virus (VZV, *n* = 5), herpes simplex virus (HSV) I or II (*n* = 4), enterovirus (*n* = 4), influenza A (*n* = 1), Toscana virus (*n* = 1) and *Borrelia burgdorferi* (*n* = 1). The etiology was unknown in 30% (*n* = 15) of patients, and in 3 of these cases, a diagnosis of autoimmune etiology was made after inclusion to the study until follow-up consultation (granulomatosis with polyangiitis, neurosarcoidosis, Bickerstaff encephalitis). For the follow up examination, patients with autoimmune or bacterial cause of disease were excluded from the analysis (Figure 1). Three patients refused to continue, three patients were lost for follow up, and 1 patient had died unrelated to the meningoencephalitis. None of the patients who were included in the study after the start of the SARS-CoV-2 pandemic tested positive for the SARS-CoV-2 virus by PCR in the acute phase.

The most important laboratory, MRI and EEG findings are presented in Table 1. Pathological findings were present in the MRI of 39 patients (leptomeningeal/plial enhancement *n* = 28, other lesions *n* = 12), thereof 15 patients had a follow-up MRI examination. The MRI showed a good recovery in 53%, who had only a few some residual lesions or none at all. An EEG was performed in 21 patients of whom 19 had pathological findings, including general slowing in 15 patients and focal lesions in 14 patients. As shown in Table 1, the most common signs and symptoms in the acute phase were headache (88%), fever (72%), and cognitive impairment such as psychomotor slowing, confusion, disorientation, and memory or concentration deficits in 68% of all patients. Overall, 15 patients (30%) had reduced consciousness, 14 were admitted to an intensive or intermediate care unit for a median of 3 days and only 3 patients required mechanical ventilation: one TBE patient for 1 day due to lung edema and 2 HSV1 patients for 1 and 3 days due to status epilepticus. The second patient with HSV1 encephalitis required intensive care for 22 days due to non-convulsive status epilepticus and severe secondary complications with hemorrhagic transformation of extensive cerebral lesions requiring surgical treatment, despite being mechanically ventilated for only 3 days. This patient had an underlying diagnosis of multiple sclerosis and was under treatment with fingolimod. In clinical routine follow-up 6 months after the acute disease, the patient remained unable to work due to severe neurocognitive deficits. Unfortunately, the patient refused to participate further in the follow-up study visits due to language difficulties and the need to travel a long distance to our hospital.

**Table 1:** Demographic and clinical patient data.

	Total	TBE	Other viral*	Unknown
**Number**	50	16	15	15
Age**	43 [32, 62]	48 [40, 65]	35 [31, 45]	52 [32, 73]
Sex (male)***	32 (64)	12 (75)	11 (73)	7 (47)
**Diagnosis**				
Encephalitis	6 (12)	0 (0)	3 (20)	1 (7)
Meningoencephalitis	29 (58)	15 (94)	4 (27)	8 (53)
Meningitis	15 (30)	1 (6)	8 (53)	6 (40)
**Severity**				
Mild	16 (32)	4 (25)	7 (47)	4 (27)
Moderate	22 (44)	8 (50)	4 (27)	7 (47)
Severe	12 (24)	4 (25)	4 (27)	4 (27)
**Headache**	44 (88)	15 (94)	13 (87)	13 (87)
**Neck pain**	18 (36)	4 (25)	8 (53)	5 (33)
**Fever**	36 (72)	15 (94)	10 (67)	11 (73)
**Vomiting/nausea**	27 (54)	10 (63)	7 (47)	7 (47)
**Phono−/photophobia**	15 (30)	3 (19)	7 (47)	3 (20)
**Arthralgia**	7 (14)	1 (6)	3 (20)	3 (20)
**Myalgia**	7 (14)	5 (31)	2 (13)	0 (0)
**Stomach pain/diarrhea**	5 (10)	4 (25)	1 (7)	0 (0)
**Cranial nerve dysfunction**	10 (20)	3 (19)	3 (20)	2 (13)
**Aphasia/dysarthria**	20 (40)	12 (75)	3 (20)	5 (33)
**Motor deficit**	13 (26)	7 (44)	2 (13)	4 (27)
**Balance/coordination deficit**	23 (46)	11 (69)	5 (33)	5 (33)
**Abnormal behavior or psychiatric disorder**	11 (22)	5 (31)	2 (13)	3 (20)
**Cognitive impairment**	34 (68)	14 (88)	7 (47)	9 (60)
Psychomotor slowing	21 (62)	13 (93)	4 (57)	4 (44)
Confusion/disorientation	20 (59)	9 (64)	4 (57)	5 (56)
Memory/concentration deficit	24 (71)	11 (79)	4 (57)	6 (67)
**Epileptic seizure**	7 (14)	1 (6)	4 (27)	1 (7)
Status epilepticus	2 (29)	0 (0)	2 (50)	0 (0)
**Reduced vigilance**	12 (24)	6 (38)	4 (27)	2 (13)
GCS	8.5 [7.5,12]	11 [8.0,13]	7.5 [6.0,10]	8.5 [8.0,9.0]
Mechanical ventilation	3	1^#^	2^#^	0
**ICU/IMC treatment**	14 (28)	5 (31)	4 (27)	5 (33)
**Acute sleep wake disorder**	33 (66)	14 (88)	9 (60)	7 (47)
**Median days in hospital**	8.0 [4.0, 12]	10 [6.0, 13]	5.0 [3.0, 9.0]	8.0 [6.0, 11]
**Discharge to**				
Home	30 (60)	5 (31)	12 (80)	10 (67)
Rehabilitation Unit	16 (32)	10 (63)	2 (13)	3 (20)
Other hospital	4 (8)	1 (6)	1 (7)	2 (13)
**Paraclinical**				
**C-reactive protein mg/l**	6.5 [3.0, 20]	6.0 [3.0, 16]	5.0 [3.0, 8.0]	23 [3.0, 79]
**Leucocytes g/l**	10 [6.6, 12]	11 [7.3, 15]	7.0 [6.2, 10]	10 [6.8, 12]
**Cerebrospinal fluid (CSF)**				
Cell count /m	152 [55, 241]	105 [72, 178]	250 [60, 457]	155 [42, 232]
Protein g/l	0.72 [0.50, 1.2]	0.74 [0.63, 1.1]	0.76 [0.46, 1.8]	0.51 [0.45, 1.2]
Lactat mmol/l	2.5 [2.0, 3.1]	2.3 [1.9, 2.8]	2.3 [2.1, 3.5]	2.8 [2.0, 3.4]
Albumin quotient	14 [7.4, 22]	15 [13, 22]	11 [7.1, 27]	11 [6.9, 20]
OCBs analysis /pathological	28 (56)/12 (43)	8 (50)/2 (25)	7 (47)/4 (57)	9 (60)/4 (44)
Typ II; III; IV; V	3; 1; 7; 1	0; 1; 1; 0	1; 0; 2; 1	1; 0; 3; 0
**MRI performed/pathological**	46 (92)/39 (85)	15 (94)/10 (66)	13 (86)/11 (85)	14 (93)/14 (100)
Leptomeningeal, pial enhancement	28 (72)	7 (70)	7 (64)	12 (86)
**EEG performed/pathological**	21 (42)/19 (90)	8 (50)/8 (100)	5 (33)/4 (80)	7 (46)/6 (86)

Data are in n (%) for categorical data and median [IQR] for continuous data. Patients may present more than one complaint within a symptom group. TBE, tick-borne encephalitis. VZV, varicella zoster virus. HSV, herpes simplex virus. ICU, intensive care unit. IMC, intermediate care unit. OCBs, oligoclonal bands. Type II: oligoclonal IgG bands in CSF, type III: oligoclonal bands in CSF and serum with additional bands in CSF, type IV: identical oligoclonal bands in CSF and serum, type V: monoclonal. *Other viral: VZV (*n* = 5), HSV I or II (*n* = 4), Enterovirus (*n* = 4), influenza A (*n* = 1), Toscana virus (*n* = 1). **p = 0.11, ***p = 0.2.

# Mechanical ventilation in FSME patient for 1 day (lung edema) and in 2 HSV1 patient for 1 and 3 days (status epilepticus).

Seven (14%) patients had at least one epileptic seizure, only 2 patients had a status epilepticus (both had HSV1).

### 3.1 Cognitive testing, scores and follow-up results

The first follow-up took place a median of 8 months (range 6–17 months) and the telephone call follow-up a median of 20 months (range 14–38 months) after hospital discharge. At the first and second follow-up, only 23 and 33% of patients, respectively, reported the absence of any remaining signs or symptoms, as shown in Table 2. The median Barthel index was 100 (score < 100 *n* = 12, lowest score 45) at discharge and 100 (score < 100 *n* = 1) at the second follow-up consultation, reflecting patients who were not severely physically impaired.

**Table 2:** Signs and symptoms at follow ups.

	8 months					20 months				
	Total	Mild	Moderate	Severe	*p*-value	Total	Mild	Moderate	Severe	*p*-value
**N**	39	14	14	11		39	14	14	11	
**Free of complaints**	9 (23)	4 (29)	3 (21)	2 (18)	0.90	13 (33)	5 (36)	5 (36)	3 (27)	1.00
**Headache (< 15d/month)**	10 (26)	4 (29)	3 (21)	3 (27)	1.00	5 (13)	3 (21)	1 (7)	1 (9)	0.60
**Cognitive impairment**	18 (46)	5 (36)	7 (50)	6 (55)	0.72	14 (36)	3 (21)	5 (36)	6 (55)	0.22
**EDS/fatigue**	22 (56)	7 (50)	9 (64)	6 (55)	0.79	13 (33)	3 (21)	7 (50)	3 (27)	0.32
Preexisting	6 (27)	3 (43)	2 (22)	1 (17)	0.59	1 (8)	0 (0)	1 (14)	0 (0)	1.00
Newly appeared	16 (73)	4 (57)	7 (78)	5 (83)	0.59	12 (92)	3 (100)	6 (86)	3 (100)	1.00
**Sleep disorder**	17 (44)	5 (36)	6 (43)	6 (55)	0.66	17 (44)	6 (43)	7 (50)	4 (36)	0.92
Preexisting	11 (65)	2 (40)	5 (83)	4 (67)	0.38	5 (29)	2 (33)	2 (29)	1 (25)	1.00
Newly appeared	6 (35)	3 (60)	1 (17)	2 (33)	0.38	12 (71)	4 (67)	5 (71)	3 (75)	1.00
**Same performance as before**	19 (66)	12 (86)	4 (36)	3 (75)	0.028	25 (76)	12 (92)	8 (73)	5 (56)	0.15
Still physically impaired	4 (14)	0 (0)	3 (27)	1 (25)	0.08	2 (6)	0 (0)	0 (0)	2 (22)	0.07
Still mentally impaired	6 (21)	2 (14)	4 (36)	0 (0)	0.37	6 (18)	1 (8)	3 (27)	2 (22)	0.53
Still physically and mentally impaired	10 (26)	0 (0)	3 (21)	7 (64)	<0.001	6 (15)	1 (7)	3 (21)	2 (18)	0.64
**More quickly exhausted**	20 (51)	6 (43)	7 (50)	7 (64)	0.66	19 (49)	7 (50)	7 (50)	5 (45)	1.00
Mentally	15 (75)	6 (100)	6 (86)	3 (43)	0.08	10 (53)	3 (43)	5 (71)	2 (40)	0.60
Physically	1 (5)	0 (0)	1 (14)	0 (0)	1.00	1 (5)	1 (14)	0 (0)	0 (0)	1.00
Both	4 (20)	0 (0)	0 (0)	4 (57)	0.018	8 (42)	3 (43)	2 (29)	3 (60)	0.84
**Limitation in social life**	15 (38)	2 (14)	7 (50)	6 (55)	0.09	13 (33)	2 (14)	6 (43)	5 (45)	0.18
Slight	9 (60)	2 (100)	3 (43)	4 (67)	0.48	13 (100)	2 (100)	6 (100)	5 (100)	0.19
Severe	6 (40)	0 (0)	4 (57)	2 (33)	0.48	0 (0)	0 (0)	0 (0)	0 (0)	0.20
**Limitation in professional life**	8 (21)	1 (7)	5 (36)	2 (18)	0.17	11 (28)	3 (21)	4 (29)	4 (36)	0.90
Slight	4 (50)	1 (100)	2 (40)	1 (50)	0.18	10 (91)	3 (100)	4 (100)	3 (75)	0.91
Severe	4 (50)	0 (0)	3 (60)	1 (50)	0.19	1 (9)	0 (0)	0 (0)	1 (25)	0.92
**mRS (mean)**	1.1 (0.81)	0.71 (0.47)	1.2 (0.89)	1.4 (0.92)	0.10	0.82 (0.64)	0.64 (0.50)	1.0 (0.78)	0.82 (0.60)	0.35
**mRS (median)**	1.0 [1.0, 2.0]	1.0 [0.00, 1.0]	1.0 [1.0, 2.0]	1.0 [1.0, 2.0]	0.11	1.0 [0.0, 1.0]	1.0 [0.0, 1.0]	1.0 [1.0, 1.0]	1.0 [0.0, 1.0]	0.45
**GOS (mean)**	–	–	–	–	–	4.9 (0.26)	5.0 (0.0)	4.9 (0.27)	4.8 (0.40)	0.25
**GOS (median)**	–	–	–	–	–	5.0 [5.0, 5.0]	5.0 [5.0, 5.0]	5.0 [5.0, 5.0]	5.0 [5.0, 5.0]	0.25
**Eq-5D-5L**	0.89 (0.13)	0.91 (0.09)	0.90 (0.08)	0.84 (0.20)	0.40	0.93 (0.07)	0.95 (0.06)	0.92 (0.05)	0.90 (0.10)	0.21
**Sf-36**	74 (16)	81 (11)	77 (10)	64 (22)	0.033	76 (14)	82 (11)	78 (12)	68 (17)	0.05

Data are in n (%) for categorical data and in mean (sd) or median [IQR] for continuous data.

**Figure 2:**
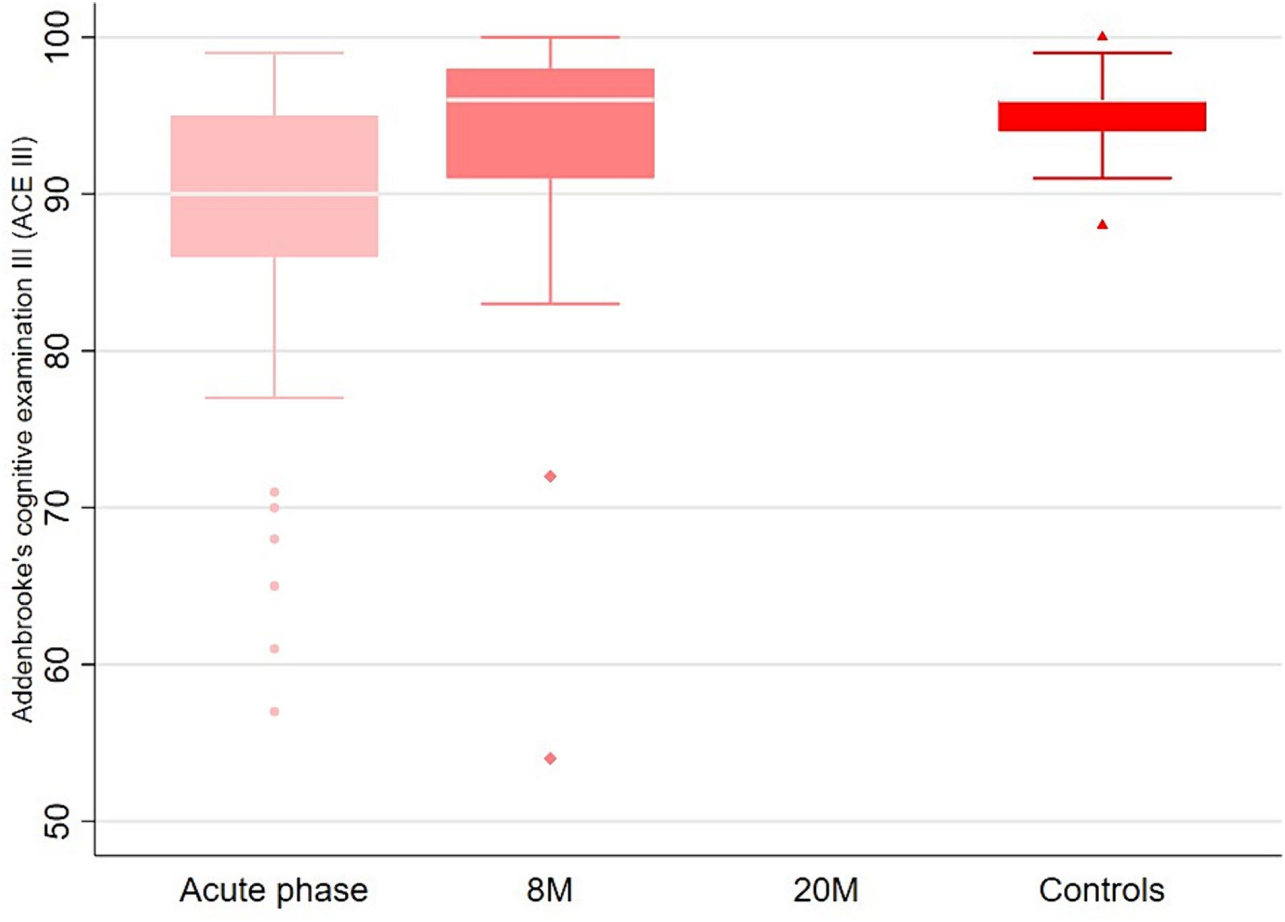
Comparison of the ACE III scores between the acute phase, follow up at 8 months and the controls.

At the first and second follow-up, 46% (*n* = 18) and 36% (*n* = 14) of patients, respectively, reported the persistence of subjective memory or concentration deficits (Table 2). However the objective testing of the ACE III showed a median total score of 90 (IQR 86, 95) in the acute phase and 96 (IQR 91, 98) at 8 months and 96 (IQR 94, 96) in the control group (Figure 2). Headache, EDS/fatigue and sleep disturbances were the most frequent long-term sequelae besides cognitive impairment (Table 2), and the numbers decreased over time (except for the sleep problems). At the telephone follow-up, 24% still (*n* = 14) reported being subjectively less overall performant than before the disease and 49% (*n* = 19) reported being more quickly exhausted (mentally and/or physically). At the second follow-up, 64% still had persisting signs and symptoms, but only 32% felt subjectively slightly affected by these sequelae in social life and 28% in their professional life (91% slightly, 1 patient severely). When asked directly, none of the patients reported a recalled or more severe COVID-19 infection in the meantime. However, neither PCR nor serological tests were carried out in this regard at any follow-up.

**Figure 3:**
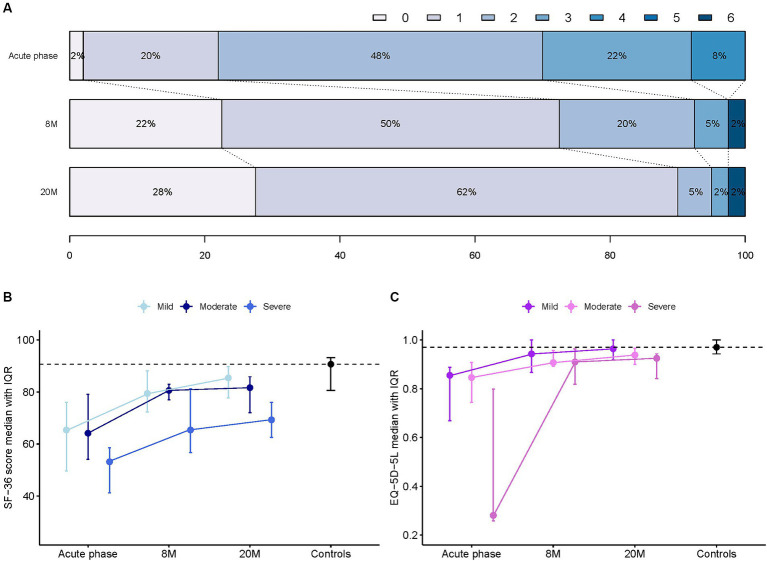
Outcome data. **(A)** Comparison of the level distributions of the modified ranking scale (mRS) between the acute phase and follow up times. 0 = No symptoms, 1 = No significant disability, 2 = Slight disability, 3 = Moderate disability, 4 = Moderately severe disability, 5 = severe disability, 6 = dead. **(B)** Comparison of sf-36 scores according to the severity grade during the acute phase, the follow up times and the controls. **(C)** Comparison of EQ-5D-5L scores according to the severity during the acute phase, the follow up times and the controls.

**Figure 4:**
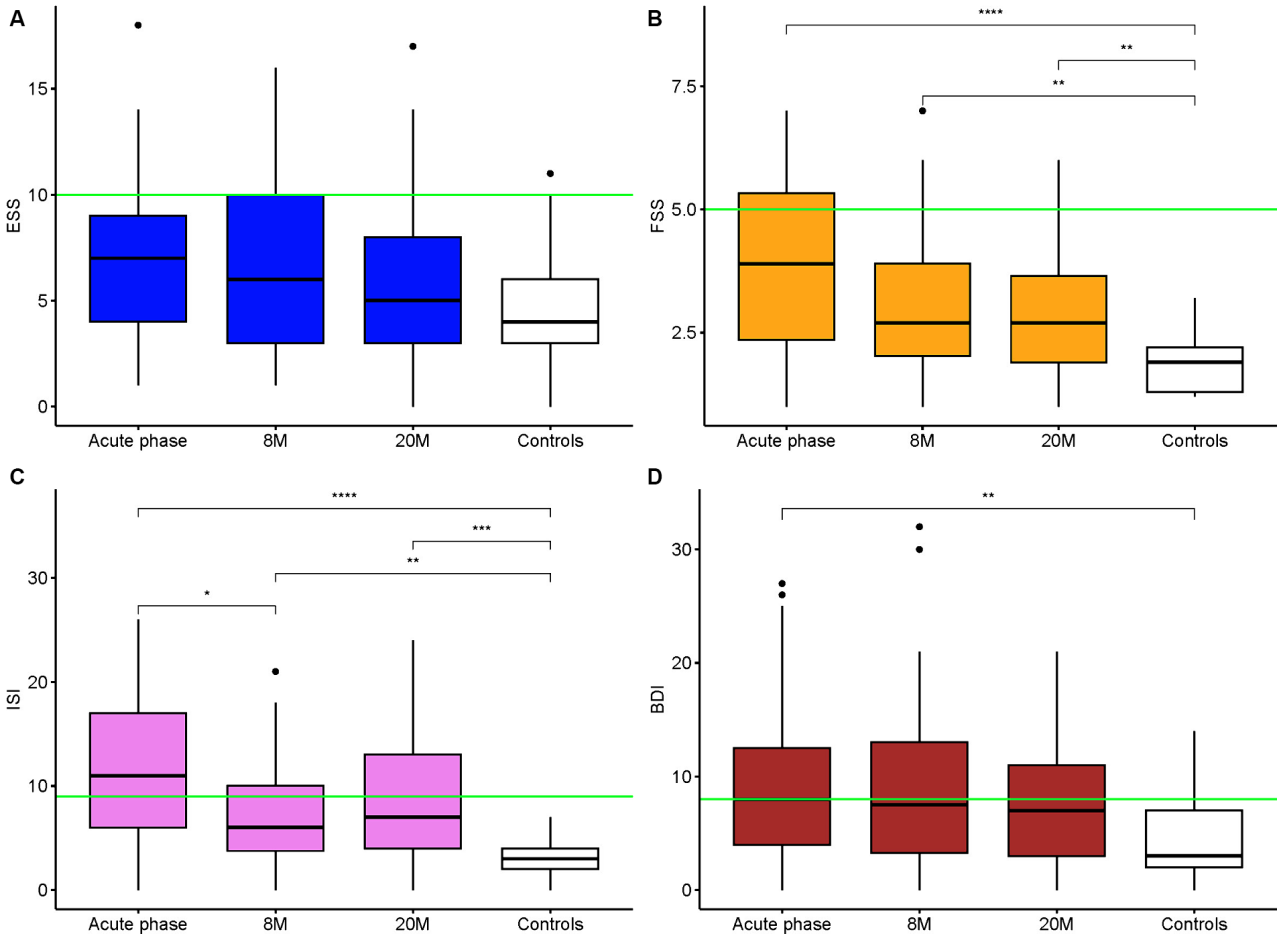
Epworth Sleepiness Scale, Fatigue Severity Scale, Insomnia Severity Index and Beck Depression Inventory II Scores Comparison of the ESS **(A)**, FSS **(B)**, ISI **(C)**, BDI II **(D)** scores between the acute phase, the follow up times and the controls. The green lines represent the pathological cutoff score. **p-values <0.01 (for us 0.008333 is the adjusted threshold), ***p-values <0.001, ****p-values <0.0001.

The responses to the health questionnaires, sf-36 and EQ-5D-5L, showed an improvement of the scores at the follow-up assessments compared to the acute phase. However, the scores remained generally lower than those of the healthy controls (*p* = 0.003 for sf-36 score; Figure 3). ESS, FSS, ISI and BDI II scores are shown in Figure 4. Details of clinical follow-up data are provided in Supplementary Tables S1–S5.

The actigraphy data showed that more than half of the study participants had poor sleep hygiene and about one third had irregular bedtimes; note that patients and controls showed a similar prevalence. Frequent inactivity phases could only be observed during the acute phase and none of the controls showed fragmented sleep or low amplitude activity at night. During the acute phase, patients had a significantly higher inactivity index and more time in bed than at follow-up and compared to healthy controls (Table 3).

**Table 3:** Sleep actigraphy.

	Acute phase	8 months	Controls	*p*-value
N	50	39	21	
Normal	6 (12)	9 (23)	8 (38)	0.045
Poor sleep hygiene	26 (52)	18 (46)	11 (52)	0.88
Irregular lay time	17 (34)	12 (31)	7 (33)	0.96
Fragmented sleep	10 (20)	4 (10)	0 (0)	0.05
Frequent inactivity phases	19 (38)	0 (0)	0 (0)	<0.001
Relative sleep insufficiency	0 (0)	2 (5)	0 (0)	0.16
Low tension activity at night	6 (12)	5 (13)	0 (0)	0.24
Inactivity index (%)	48 (10)	39 (7.5)	35 (4.9)	<0.001
Time in bed (h)	8.9 (0.77)	8.2 (1.1)	7.9 (0.62)	<0.001
Sleep efficiency (%)	80 (10)	83 (7.4)	80 (7.2)	0.24

Data are in n (%) for categorical data and in mean (sd) for continuous data.

### 3.2 Outcome predictors

When applying a logistic model with mRS and limitations in social/professional life dichotomized as outcome (mRS = 0 vs. mRS > 0 and no limitations vs. limitations) no predictors for the outcome could be identified (Supplementary Table S6).

Another aim of the study was to assess the well-being of the individuals, particularly with regard to limitations in their social and/or professional life. No specific symptoms correlating with restrictions in their social/professional domain (Supplementary Tables S7, S8) were identifiable. Patients with a follow-up later within our time frame did not differ in outcome parameters compared to patients examined earlier after recovering from the acute disease.

## 4 Discussion

The most frequent causative agent of meningoencephalitis and meningitis was TBE, found in one third of the study population, whereas in another third of patients, the cause remained unknown. Two years after the acute disease, the overall outcome of the study patients was not impaired, when measured by crude assessments such as mRS (median 1, IQR 1, 1) or the ability to return to work at the previous level (93%) and was not associated with disease severity in the acute phase. However, subjectively, only 33% of patients reported that they were free of any specific signs or symptoms and 76% felt completely recovered after a median of 2 years. The most important persisting signs and symptoms were subjective cognitive impairment (36%), fatigue and/or EDS (31%), disturbed nighttime sleep (31%) and headaches (13%). Almost half the patients reported feeling more rapidly exhausted after cognitive effort (53%). However, these signs and symptoms had only a relatively mild impact on the patients’ social and/or professional life, affecting only a third of patients. This is reflected in the sf-36, the FSS and ISI questionnaire scores, which improved over time but remained significantly different from those of the healthy controls. The ACE scores showed a favorable cognitive recovery, underlining the relatively mild nature of self-reported cognitive impairments. In line with our hypothesis, these long-term outcome findings were not associated with the disease severity in the acute phase and also occurred in patients with “mild” viral meningitis.

Our findings on patients’ outcome are well in line with other studies, although the numbers of long-term sequelae vary considerably between the different study designs and study populations. For instance, studies on TBE patients described post-infectious sequelae in 19–33% of the patients (12, 14, 27). A study investigating the outcome after HSV encephalitis reported sequelae in 69% (28) and a study from France on infectious encephalitis with various causes described sequelae in 61% (2). In our own study in Switzerland, neurological complaints after non-bacterial meningitis were reported in 42% of patients (5) and a study from Germany reported that 40% of patients had mild to moderate cognitive impairment after viral meningitis (15).

In contrast to a large study on viral meningitis in the United Kingdom (UK) our patients did not show significantly lower Eq-5D-5L scores at follow-up, which may be explained by our small sample size (4). It is noteworthy that our study population included mostly mildly or moderately severely ill patients, and due to the study design (inclusion criterion of independently signed informed consent), very severe cases were not eligible. This explains why only 28% of our patients were admitted to an intensive or intermediate care unit and only 3 patients needed short term mechanical ventilation. Thus it is surprising that—despite improvements over time—67% of the patients nevertheless reported persistent residual signs and symptoms after a median of 2 years. In a study on long-term outcome after TBE in central Europe, the frequency of post-encephalitic syndrome diminished over time, stabilizing 12 months after the acute illness, whereas the severity continued to decline over a period of 2–7 years after infection (27). This implies that patients still experiencing sequelae after more than 1 year are unlikely to recover completely, but there is still potential for a reduction in the severity and number of complaints.

Post-acute infection syndromes (PAISs), which can be caused by various infectious agents, have been known for a long time, but are still largely unexplained and understudied. Since the COVID-19 pandemic, this phenomenon has gained more attention, as these long-term sequalae, also known as “long COVID,” became a recurring post-acute infection syndrome (29). Its reported prevalence varies substantially, depending on the study profile (e.g., different severity grades, follow-up periods, virus variants). For example, a study from the UK reported a prevalence of 4.5% among outpatients with the omicron virus (30), whereas another study from the UK looking at hospitalized patients reported a prevalence of 55% (31). Primary symptoms in patients with PAISs included an overall poor functional status, exertion intolerance, chronic fatigue that is not relieved by sleep or rest, neurocognitive and sensory impairments, dysautonomia, musculoskeletal complaints, flu-like symptoms, and other feelings of illness. Irritability, mood swings, and signs of depression, as well as a wide range of other nonspecific neurological and immunological symptoms are frequently present (29).

Multiple potential explanations exist for the pathogenesis of PAISs. One possible mechanism might include chronic stimulation of the immune system. Another hypothesis is based on immune activation, which involves targeting self-antigens. The possibility of inflammation-triggered changes leading to tissue dysfunction and damage has also been discussed (29).

Unlike in bacterial meningitis, where the outcome is associated with the type of pathogen (32), no predictive factors related to the pathogens could be found in viral meningitis. This could suggest that, in viral meningitis, the host immune response plays a more significant role in the recovery process than in bacterial meningitis (33). An experimental study described how HSV-1 brain infection induces neuroimmune responses, which persist beyond the presence of detectable virus replication (34). Due to the meninges’ ability to promote strong inflammatory responses, infection of this protective compartment, whether acute or chronic, may lead to considerable neurological dysfunction (35).

In recent years, the incidence of TBE in Switzerland has increased (36), making it the most important viral cause of meningoencephalitis and meningitis (5). There are several possible reasons for this, including the expansion of the tick endemic areas and/or more people engaging in outdoor activities (36). In our cohort, no vaccine breakthrough cases could be observed. In total, only 4 patients had full and 3 incomplete vaccination status, which is much less than the mean Swiss coverage (33% in 2018) (37). Improving the vaccine coverage in Switzerland could be a key target in TBE prevention. Patients with TBE showed a comparable clinical presentation (signs and symptoms, laboratory and imaging findings) to other etiologies in the acute phase as well as the outcome. These findings align with a large study from Denmark, which also reported the outcome in viral meningitis patients to be similar, regardless of the underlying etiology, including cases where no specific pathogen was identified (33).

To our knowledge, no actigraphy analysis during and after meningitis or meningoencephalitis has been done so far. This method provides additional information to complement the sleep questionnaires and is a less invasive and costly procedure than polysomnography (PSG). As expected, during hospitalization, a large number of patients had frequent inactivity phases during the day, and fragmented sleep, as well as the highest inactivity index and time in bed. During the acute illness, the actigraphy analysis revealed a poorer sleep quality, but there were no relevant differences in sleep patterns between patients in the follow-up period and the control group. Even though 44% of the patients reported having sleep problems (35% of these newly appeared after the disease) after a mean of 8 months after the disease, the sleep actigraphy analysis did not find significant differences compared to the control group. Nevertheless, the inactivity index and time spent in bed by the controls was lower than in the patients’ follow-up actigraphy (statistically not significant), suggesting that individuals recovering from such a disease may require additional sleep and daytime recuperation even after 8 months.

One study focusing on the sleep architecture compared the PSG of patients with a history of TBE and controls. The authors observed a difference in the scores of the Functional Outcome of Sleep Questionnaire but could not reproduce the difference in the PSG characteristics (38). A study focusing on wake–sleep behavior reported that 27% of viral meningitis patients had sleep disturbances (17), which aligns well with the 31% in our study population.

One limitation of our study is the small number of patients, which was primarily due to the difficulty of recruiting patients during the COVID-19 pandemic. The pandemic also had an impact on the subsequent assessments, which explains the variations in the duration of follow-up. Another limitation is the maximum follow-up time with a mean of 20 months, which may not capture longer-term outcomes beyond that timeframe. Another limitation is the suboptimal control group of healthy controls. A control group of patients with a monophasic infectious disease, treated in hospital without affection of the central nervous system would have been much more adequate. Unfortunately, this would have meant an insurmountable personnel and logistical effort four our own academic study. In addition, it was not easy for us to identify an ideal control group with the same epidemiological profile. In regard to limitations we want to mention possible biases and our effort to address these potential sources of biases. Since all patients had to sign the informed consent themselves, this naturally represents a source of selection bias. In the inclusion of patients but also regarding the time period when neurocognitive testing was feasible. However, regarding inclusion of patients this was not a relevant problem for our study, as the main aim was to investigate the clinical course and outcome of the less severely affected patients. With regard to the follow-up examinations, a recall bias must be mentioned, which we tried to compensate for with the most standardized survey possible. Regarding confounders and effect modifiers that may influence the outcome, other self-limiting infectious diseases such as a COVID-19 disease that has occurred in the meantime must be mentioned. After a COVID-19 disease, the same signs and symptoms may occur as our patients reported. When asked directly about this, none of our patients stated that they had been ill with COVID-19 since their hospital stay.

Since the spectrum of pathogens causing meningitis and meningoencephalitis can vary greatly from region to region, our results can only be generalized to a limited extent. Our findings are particularly relevant for European regions with a similar spectrum of pathogens and TBE as the most common viral cause of meningoencephalitis.

In conclusion, independent of the acute disease severity, and despite constant recovery over time, a significant proportion of the patients presented long-term sequelae after a median of 2 years after surviving acute non-bacterial meningitis, meningoencephalitis or encephalitis. The most important complaints were subjective cognitive impairment, headache, subjective feeling of reduced cognitive performance and disturbances in the sleep–wake rhythm. Nevertheless, only one third of patients reported that they still had mild impairments in their social and/or professional life due to the long-term sequelae. There was no significant difference in the actigraphy analysis after 8 months between the patients and the control group, although the FSS and ISI scores remained higher compared to healthy controls.

Future research could concentrate on sleep patterns using actigraphy in a larger study population. Furthermore, focusing on biomarkers correlating with long-term sequelae could help to understand PAISs after meningitis or meningoencephalitis and could be a basis for future treatments.

## Data availability statement

The raw data supporting the conclusions of this article will be made available by the authors, without undue reservation.

## Ethics statement

The studies involving humans were approved by Kantonale Ethikkommission Bern, Schweiz. The studies were conducted in accordance with the local legislation and institutional requirements. The participants provided their written informed consent to participate in this study.

## Author contributions

JS: Formal analysis, Investigation, Visualization, Writing – original draft. MB: Data curation, Formal analysis, Software, Writing – review & editing. AB: Investigation, Writing – review & editing. LA: Investigation, Writing – review & editing. FS-R: Investigation, Project administration, Writing – review & editing. SL: Conceptualization, Writing – review & editing. AD: Conceptualization, Data curation, Funding acquisition, Investigation, Project administration, Resources, Supervision, Writing – review & editing.
