# Supplementary material for: Long-term sequelae after viral meningitis and meningoencephalitis are frequent, even in mildly affected patients, a prospective observational study
Source: Front Neurol. 2024 Jul 17;15:1411860. doi: 10.3389/fneur.2024.1411860 (PMC11288970; doi:10.3389/fneur.2024.1411860)
Supplement: Supplementary file 1 [file Table_1.DOCX]

| Table S1 | 8 months | | | | 20 months | | | |
| --- | --- | --- | --- | --- | --- | --- | --- | --- |
|  | **Total**  **(N = 39)** | **TBE**  **(N = 15)** | **Unknown**  **(N = 11)** | **Others (viral)**  **(N = 13)** | **Total**  **(N = 39)** | **TBE**  **(N = 15)** | **Unknown**  **(N = 11)** | **Others (viral)**  **(N = 13)** |
| Free of complaints | 9 (23) | 2 (13) | 2 (18) | 5 (38) | 13 (33) | 2 (14) | 5 (42) | 6 (46) |
| Headache (< 15d/month) | 10 (26) | 1 (7) | 5 (45) | 4 (31) | 5 (13) | 0 (0) | 3 (25) | 2 (15) |
| Cognitive impairment | 18 (46) | 7 (47) | 7 (64) | 4 (31) | 14 (36) | 7 (50) | 4 (33) | 3 (23) |
| Cognitive deficits | 7 (18) | 3 (20) | 3 (27) | 1 (8) | 2 (5) | 1 (7) | 0 (0) | 1 (8) |
| Memory/attention disorder | 17 (44) | 7 (47) | 6 (55) | 4 (31) | 14 (36) | 7 (50) | 4 (33) | 3 (23) |
| Confusion/disorientation | 1 (3) | 0 (0) | 1 (9) | 0 (0) | 0 (0) | 0 (0) | 0 (0) | 0 (0) |
| Cranial nerve disorder | 1 (3) | 0 (0) | 0 (0) | 1 (8) | 1 (3) | 0 (0) | 0 (0) | 1 (8) |
| Aphasia | 3 (8) | 1 (7) | 0 (0) | 2 (15) | 3 (8) | 0 (0) | 0 (0) | 3 (23) |
| Epileptic seizures | 1 (3) | 0 (0) | 0 (0) | 1 (8) | 0 (0) | 0 (0) | 0 (0) | 0 (0) |
| Focal motoric deficits | 3 (8) | 3 (20) | 0 (0) | 0 (0) | 3 (8) | 3 (21) | 0 (0) | 0 (0) |
| Focal sensory deficits | 1 (3) | 1 (7) | 0 (0) | 0 (0) | 2 (5) | 1 (7) | 1 (8) | 0 (0) |
| Gait disorder | 3 (8) | 2 (13) | 1 (9) | 0 (0) | 2 (5) | 1 (7) | 1 (8) | 0 (0) |
| Micturition disorder | 1 (3) | 1 (7) | 0 (0) | 0 (0) | 0 (0) | 0 (0) | 0 (0) | 0 (0) |
| Psychiatric symptoms | 3 (8) | 2 (13) | 0 (0) | 1 (8) | 2 (5) | 2 (14) | 0 (0) | 0 (0) |
| Excessive fatigue/daytime sleepiness | 22 (56) | 8 (53) | 5 (45) | 9 (69) | 13 (33) | 5 (36) | 4 (33) | 4 (31) |
| Preexisting | 6 (27) | 1 (13) | 0 (0) | 5 (56) | 1 (8) | 0 (0) | 0 (0) | 1 (25) |
| Tired but cannot sleep | 7 (32) | 2 (25) | 3 (60) | 2 (22) | 0 (0) | 0 (0) | 0 (0) | 0 (0) |
| Low on energy and exhausted. | 6 (27) | 2 (25) | 3 (60) | 1 (11) | 10 (77) | 4 (80) | 3 (75) | 3 (75) |
| Powerless and listless | 4 (18) | 1 (13) | 2 (40) | 1 (11) | 1 (8) | 0 (0) | 1 (25) | 0 (0) |
| Naps during the day | 9 (41) | 3 (38) | 1 (20) | 5 (56) | 1 (8) | 0 (0) | 0 (0) | 1 (25) |
| Fall asleep unintentionally | 3 (14) | 0 (0) | 0 (0) | 3 (33) | 1 (8) | 0 (0) | 1 (25) | 0 (0) |
| Exhausted much faster | 5 (23) | 2 (25) | 2 (40) | 1 (11) | 4 (31) | 2 (40) | 1 (25) | 1 (25) |
| Sleep disorder | 17 (44) | 9 (60) | 4 (36) | 4 (31) | 17 (44) | 10 (71) | 4 (33) | 3 (23) |
| Preexisting | 11 (65) | 6 (67) | 2 (50) | 3 (75) | 5 (29) | 2 (20) | 2 (50) | 1 (33) |
| Disturbed falling asleep | 2 (12) | 0 (0) | 2 (50) | 0 (0) | 6 (35) | 2 (20) | 2 (50) | 2 (67) |
| Awakening during night | 12 (71) | 7 (78) | 2 (50) | 3 (75) | 7 (41) | 4 (40) | 2 (50) | 1 (33) |
| Awakening earlier than intended | 3 (18) | 3 (33) | 0 (0) | 0 (0) | 2 (12) | 2 (20) | 0 (0) | 0 (0) |
| Not feeling recovered | 2 (12) | 0 (0) | 1 (25) | 1 (25) | 3 (18) | 2 (20) | 1 (25) | 0 (0) |
| Superficial sleep | 1 (6) | 0 (0) | 0 (0) | 1 (25) | 2 (12) | 1 (10) | 0 (0) | 1 (33) |
| Same performance as before | 19 (66) | 6 (50) | 6 (86) | 7 (70) | 25 (76) | 8 (73) | 8 (89) | 9 (69) |
| Still physically impaired | 4 (14) | 3 (25) | 1 (14) | 0 (0) | 2 (6) | 1 (9) | 1 (11) | 0 (0) |
| Still mentally impaired | 6 (21) | 3 (25) | 0 (0) | 3 (30) | 6 (18) | 2 (18) | 0 (0) | 4 (31) |
| Still physically and mentally impaired | 12 (30) | 3 (20) | 5 (38) | 3 (25) | 7 (18) | 3 (21) | 3 (21) | 0 (0) |
| More quickly exhausted | 20 (51) | 8 (53) | 8 (73) | 4 (31) | 19 (49) | 8 (57) | 6 (50) | 5 (38) |
| Mentally | 15 (75) | 6 (75) | 7 (88) | 2 (50) | 10 (53) | 4 (50) | 2 (33) | 4 (80) |
| Physically | 1 (5) | 0 (0) | 0 (0) | 1 (25) | 1 (5) | 1 (13) | 0 (0) | 0 (0) |
| Both | 4 (20) | 2 (25) | 1 (13) | 1 (25) | 8 (42) | 3 (38) | 4 (67) | 1 (20) |
| Limited in social life | 47 (23) | 6 (40) | 5 (45) | 4 (31) | 13 (33) | 5 (36) | 4 (33) | 4 (31) |
| Slightly | 48 (23) | 4 (67) | 2 (40) | 3 (75) | 13 (100) | 5 (100) | 4 (100) | 4 (100) |
| Severily | 49 (23) | 2 (33) | 3 (60) | 1 (25) | 0 (0) | 0 (0) | 0 (0) | 0 (0) |
| Limited in professional life | 50 (23) | 2 (13) | 3 (27) | 3 (23) | 11 (28) | 5 (36) | 4 (33) | 2 (15) |
| Slightly | 51 (23) | 0 (0) | 2 (67) | 2 (67) | 10 (91) | 4 (80) | 4 (100) | 2 (100) |
| Severily | 52 (23) | 2 (100) | 1 (33) | 1 (33) | 1 (9) | 1 (20) | 0 (0) | 0 (0) |
| Data are in n (%) for categorical data and in mean (sd) for continuous data. Patients may present more than one complaint within a symptom group. | | | | | | | | |

| Table S2 | 8 months | | | | 20 months | | | |
| --- | --- | --- | --- | --- | --- | --- | --- | --- |
|  | **Total**  **(N = 39)** | **Mild**  **(N = 14)** | **Moderate**  **(N = 14)** | **Severe**  **(N = 11)** | **Total**  **(N = 39)** | **Mild**  **(N = 14)** | **Moderate**  **(N = 14)** | **Severe**  **(N = 11)** |
| Free of complaints | 9 (23) | 4 (29) | 3 (21) | 2 (18) | 13 (33) | 5 (36) | 5 (36) | 3 (27) |
| Headache (< 15d/month) | 10 (26) | 4 (29) | 3 (21) | 3 (27) | 5 (13) | 3 (21) | 1 (7) | 1 (9) |
| Cognitive impairment | 18 (46) | 5 (36) | 7 (50) | 6 (55) | 14 (36) | 3 (21) | 5 (36) | 6 (55) |
| Cognitive deficits | 7 (18) | 1 (7) | 3 (21) | 3 (27) | 2 (5) | 0 (0) | 0 (0) | 2 (18) |
| Memory/attention disorder | 17 (44) | 4 (29) | 7 (50) | 6 (55) | 14 (36) | 3 (21) | 5 (36) | 6 (55) |
| Confusion/disorientation | 1 (3) | 1 (7) | 0 (0) | 0 (0) | 0 (0) | 0 (0) | 0 (0) | 0 (0) |
| Cranial nerve disorder | 1 (3) | 0 (0) | 1 (7) | 0 (0) | 1 (3) | 0 (0) | 1 (7) | 0 (0) |
| Aphasia | 3 (8) | 0 (0) | 1 (7) | 2 (18) | 3 (8) | 1 (7) | 0 (0) | 2 (18) |
| Epileptic seizures | 1 (3) | 0 (0) | 0 (0) | 1 (9) | 0 (0) | 0 (0) | 0 (0) | 0 (0) |
| Focal motoric deficits | 3 (8) | 0 (0) | 1 (7) | 2 (18) | 3 (8) | 0 (0) | 1 (7) | 2 (18) |
| Focal sensory deficits | 1 (3) | 0 (0) | 0 (0) | 1 (9) | 2 (5) | 0 (0) | 1 (7) | 1 (9) |
| Gait disorder | 3 (8) | 0 (0) | 1 (7) | 2 (18) | 2 (5) | 0 (0) | 0 (0) | 2 (18) |
| Micturition disorder | 1 (3) | 0 (0) | 0 (0) | 1 (9) | 0 (0) | 0 (0) | 0 (0) | 0 (0) |
| Psychiatric symptoms | 3 (8) | 2 (14) | 0 (0) | 1 (9) | 2 (5) | 0 (0) | 1 (7) | 1 (9) |
| Excessive fatigue/daytime sleepiness | 22 (56) | 7 (50) | 9 (64) | 6 (55) | 13 (33) | 3 (21) | 7 (50) | 3 (27) |
| Preexistent | 6 (27) | 3 (43) | 2 (22) | 1 (17) | 1 (8) | 0 (0) | 1 (14) | 0 (0) |
| Newly appeared | 16 (73) | 4 (57) | 7 (78) | 5 (83) | 12 (92) | 3 (100) | 6 (86) | 3 (100) |
| Tired but cannot sleep | 7 (32) | 2 (29) | 3 (33) | 2 (33) | 0 (0) | 0 (0) | 0 (0) | 0 (0) |
| Low on energy and exhausted | 6 (27) | 1 (14) | 1 (11) | 4 (67) | 10 (77) | 2 (67) | 5 (71) | 3 (100) |
| Powerless and listless | 4 (18) | 0 (0) | 1 (11) | 3 (50) | 1 (8) | 0 (0) | 0 (0) | 1 (33) |
| Naps during the day | 9 (41) | 3 (43) | 4 (44) | 2 (33) | 1 (8) | 0 (0) | 1 (14) | 0 (0) |
| Fall asleep unintentionally | 3 (14) | 0 (0) | 2 (22) | 1 (17) | 1 (8) | 1 (33) | 0 (0) | 0 (0) |
| Exhausted much faster | 5 (23) | 1 (14) | 2 (22) | 2 (33) | 4 (31) | 2 (67) | 1 (14) | 1 (33) |
| Sleep disorder | 17 (44) | 5 (36) | 6 (43) | 6 (55) | 17 (44) | 6 (43) | 7 (50) | 4 (36) |
| Preexistent | 11 (65) | 2 (40) | 5 (83) | 4 (67) | 5 (29) | 2 (33) | 2 (29) | 1 (25) |
| Newly appeared | 6 (35) | 3 (60) | 1 (17) | 2 (33) | 12 (71) | 4 (67) | 5 (71) | 3 (75) |
| Disturbed falling asleep | 2 (12) | 0 (0) | 1 (17) | 1 (17) | 6 (35) | 2 (33) | 2 (29) | 2 (50) |
| Awakening during night | 12 (71) | 3 (60) | 5 (83) | 4 (67) | 7 (41) | 2 (33) | 4 (57) | 1 (25) |
| Awakening earlier than intended | 3 (18) | 1 (20) | 0 (0) | 2 (33) | 2 (12) | 1 (17) | 0 (0) | 1 (25) |
| Not feeling recovered | 2 (12) | 1 (20) | 0 (0) | 1 (17) | 3 (18) | 1 (17) | 1 (14) | 1 (25) |
| Superficial sleep | 1 (6) | 1 (20) | 0 (0) | 0 (0) | 2 (12) | 1 (17) | 1 (14) | 0 (0) |
| Same performance as before | 29 (74) | 14 (100) | 11 (79) | 4 (36) | 25 (76) | 12 (92) | 8 (73) | 5 (56) |
| Still physically impaired | 4 (14) | 0 (0) | 3 (27) | 1 (25) | 2 (6) | 0 (0) | 0 (0) | 2 (22) |
| Still mentally impaired | 6 (21) | 2 (14) | 4 (36) | 0 (0) | 6 (18) | 1 (8) | 3 (27) | 2 (22) |
| Still physically and mentally impaired | 10 (26) | 0 (0) | 3 (21) | 7 (64) | 6 (15) | 1 (7) | 3 (21) | 2 (18) |
| More quickly exhausted | 20 (51) | 6 (43) | 7 (50) | 7 (64) | 19 (49) | 7 (50) | 7 (50) | 5 (45) |
| Mentally | 15 (75) | 6 (100) | 6 (86) | 3 (43) | 10 (53) | 3 (43) | 5 (71) | 2 (40) |
| Physically | 1 (5) | 0 (0) | 1 (14) | 0 (0) | 1 (5) | 1 (14) | 0 (0) | 0 (0) |
| Both | 4 (20) | 0 (0) | 0 (0) | 4 (57) | 8 (42) | 3 (43) | 2 (29) | 3 (60) |
| Limited in social life | 15 (38) | 2 (14) | 7 (50) | 6 (55) | 13 (33) | 2 (14) | 6 (43) | 5 (45) |
| Slightly | 9 (60) | 2 (100) | 3 (43) | 4 (67) | 13 (100) | 2 (100) | 6 (100) | 5 (100) |
| Severily | 6 (40) | 0 (0) | 4 (57) | 2 (33) | 0 (0) | 0 (0) | 0 (0) | 0 (0) |
| Limited in professional life | 8 (21) | 1 (7) | 5 (36) | 2 (18) | 11 (28) | 3 (21) | 4 (29) | 4 (36) |
| Slightly | 4 (50) | 1 (100) | 2 (40) | 1 (50) | 10 (91) | 3 (100) | 4 (100) | 3 (75) |
| Severily | 4 (50) | 0 (0) | 3 (60) | 1 (50) | 1 (9) | 0 (0) | 0 (0) | 1 (25) |
| Data are in n (%) for categorical data and in mean (sd) for continuous data. Patients may present more than one complaint within a symptom group. | | | | | | | | |

| Table S3 | Acute phase (n = 50) | 8 months (n = 39) | 20 months (n = 39) | Controls (n = 21) | P-value |
| --- | --- | --- | --- | --- | --- |
| ACE III (max 100) | 87 (10) | 92 (9.3) | - | 95 (2.9) | 0.002 |
| Attention (max 18) | 17 (1.4) | 18 (0.71) | - | 18 (0.44) | 0.028 |
| Memory (max 26) | 23 (4.3) | 24 (3.9) | - | 25 (1.1) | 0.1 |
| Fluency (max 14) | 8.2 (3.6) | 11 (2.9) | - | 11 (1.9) | <0.001 |
| Language (max 26) | 24 (3.0) | 25 (2.3) | - | 26 (0.51) | 0.043 |
| Visual (max 16) | 15 (1.5) | 15 (1.2) | - | 16 (0.66) | 0.009 |
| ESS | 7.2 (3.8) | 6.5 (3.7) | 5.8 (3.7) | 4.7 (3.1) | 0.08 |
| FSS | 3.8 (1.7) | 3.0 (1.5) | 2.9 (1.3) | 1.9 (0.57) | <0.001 |
| ISI | 11 (6.8) | 7.1 (5.1) | 8.1 (5.8) | 3.0 (2.1) | <0.001 |
| BDI II | 9.3 (7.4) | 9.2 (7.9) | 7.9 (6.1) | 4.3 (3.7) | 0.038 |
| mRS (mean) | 2.1 (0.90) | 1.1 (0.81) | 0.82 (0.64) | - | <0.001 |
| mrRS (median) | 2.0 [2.0, 3.0] | 1.0 [1.0, 2.0] | 1.0 [0.00, 1.0] | - | <0.001 |
| GOS (mean) |  |  | 4.9 (0.26) |  |  |
| GOS (median) |  |  | 5.0 [5.0, 5.0] |  |  |
| Barthel index (mean) | 93 (14) | 100 (1.7) | 100 (2.4) | 100 | <0.001 |
| Barthel index (median) | 100 [100, 100] | 100 [100, 100] | 100 [100, 100] | 100 [100, 100] | <0.001 |
| Sf-36 score | 60 (20) | 74 (16) | 76 (14) | 88 (7.7) | <0.001 |
| Physical functioning | 67 (30) | 88 (20) | 91 (14) | 94 (10) | <0.001 |
| Role limitations due to physical health | 54 (41) | 77 (35) | 77 (32) | 96 (9.0) | <0.001 |
| Role limitations due to emotional problems | 66 (45) | 77 (36) | 74 (37) | 97 (10) | 0.023 |
| Energy/fatigue | 50 (24) | 55 (19) | 58 (16) | 74 (12) | <0.001 |
| Emotional well-being | 65 (23) | 71 (17) | 71 (16) | 85 (10) | 0.001 |
| Social functioning | 72 (28) | 80 (21) | 79 (23) | 93 (13) | 0.012 |
| Pain | 52 (34) | 82 (21) | 87 (18) | 92 (11) | <0.001 |
| General health | 55 (22) | 62 (21) | 65 (20) | 78 (15) | <0.001 |
| EQ-5D-5L score | 0.72 (0.26) | 0.89 (0.13) | 0.93 (0.07) | 0.96 (0.07) | <0.001 |
| Mobility | 2.1 (1.1) | 1.4 (0.96) | 1.2 (0.53) | 1.0 (0.22) | <0.001 |
| Self-care | 1.5 (0.94) | 1.1 (0.44) | 1.1 (0.24) | 1.0 (0.00) | 0.003 |
| Usual activities | 2.6 (1.5) | 1.5 (0.75) | 1.3 (0.54) | 1.0 (0.00) | <0.001 |
| Pain/discomfort | 2.5 (1.0) | 1.8 (0.81) | 1.7 (0.64) | 1.5 (0.51) | <0.001 |
| Anxiety/depression | 1.6 (0.72) | 1.6 (0.86) | 1.5 (0.7) | 1.2 (0.70) | 0.31 |
| EQ-5D-5L VAS score | 65 (19) | 80 (14) | 80 (12) | 91 (6.7) | <0.001 |
| Data are in n (%) for categorical data and in mean (sd) for continuous data. ACE III= Addenbrooke’s cognitive examination III. ESS= Epworth Sleepiness Scale. FSS=fatigue severity scale. ISI=insomnia severity index. BDI II= Beck depression Inventory II. mRS=modified ranking scale. Sf-36= short-form-36. EQ-5D-5L= EuroQoL 5-Dimension 5-Level. | | | | | |

| Table S4 | 8 months | | | | | 20 months | | | | |
| --- | --- | --- | --- | --- | --- | --- | --- | --- | --- | --- |
|  | **Total**  **(n = 39)** | **TBE**  **(n = 15)** | **Unknown**  **(n = 11)** | **Others (viral)**  **(n = 13)** | **P-value** | **Total**  **(n = 39)** | **TBE**  **(n = 15)** | **Unknown**  **(n = 11)** | **Others (viral)**  **(n = 13)** | **P-value** |
| ACE III | 92 (9.3) | 94 (4.4) | 91 (10) | 92 (14) | 0.78 |  |  |  |  |  |
| Attention | 18 (0.71) | 17 (0.88) | 18 (0.76) | 18 (0.32) | 0.35 |  |  |  |  |  |
| Memory | 24 (3.9) | 25 (1.6) | 23 (3.7) | 23 (5.8) | 0.44 |  |  |  |  |  |
| Fluency | 11 (2.9) | 11 (1.8) | 10 (3.2) | 11 (3.8) | 0.68 |  |  |  |  |  |
| Language | 25 (2.3) | 26 (0.44) | 25 (1.6) | 25 (3.8) | 0.49 |  |  |  |  |  |
| Visual | 15 (1.2) | 14 (1.2) | 15 (1.4) | 15 (0.95) | 0.12 |  |  |  |  |  |
| ESS | 6.5 (3.7) | 7.0 (4.0) | 6.4 (4.2) | 6.0 (3.1) | 0.81 | 5.8 (3.7) | 6.5 (4.5) | 5.1 (3.4) | 5.6 (3.0) | 0.66 |
| FSS | 3.0 (1.5) | 2.8 (1.5) | 4.0 (1.7) | 2.5 (0.93) | 0.05 | 2.9 (1.3) | 2.8 (1.3) | 3.2 (1.4) | 2.6 (1.3) | 0.57 |
| ISI | 7.1 (5.1) | 6.8 (5.1) | 8.8 (6.7) | 6.0 (3.3) | 0.49 | 8.1 (5.8) | 8.9 (6.5) | 9.2 (5.0) | 6.0 (5.5) | 0.36 |
| BDI II | 9.2 (7.9) | 8.1 (6.6) | 10 (9.2) | 9.5 (8.8) | 0.80 | 7.9 (6.1) | 8.6 (7.0) | 7.2 (3.8) | 7.5 (6.8) | 0.83 |
| mRS (mean) | 1.1 (0.81) | 1.3 (0.80) | 1.3 (0.90) | 0.69 (0.63) | 0.11 | 0.82 (0.64) | 1.0 (0.55) | 0.83 (0.83) | 0.62 (0.51) | 0.31 |
| mRS (median) | 1.0 [1.0, 2.0] | 1.0 [1.0, 2.0] | 1.0 [1.0, 2.0] | 1.0 [0.00, 1.0] | 0.11 | 1.0 [0.00, 1.0] | 1.0 [1.0, 1.0] | 1.0 [0.00, 1.0] | 1.0 [0.00, 1.0] | 0.23 |
| Barthel index (mean) | 100 (1.7) | 100 (0.00) | 99 (3.2) | 100 (0.00) | 0.29 | 100 (2.4) | 100 (0.00) | 99 (4.5) | 100 (0.00) | 0.30 |
| Barthel index (median) | 100 [100, 100] | 100 [100, 100] | 100 [100, 100] | 100 [100, 100] | 0.29 | 100 [100, 100] | 100 [100, 100] | 100 [100, 100] | 100 [100, 100] | 0.29 |
| Sf-36 total score | 74 (16) | 78 (16) | 63 (18) | 80 (10) | 0.039 | 76 (14) | 75 (19) | 75 (8.2) | 79 (12) | 0.83 |
| Physical functioning | 88 (20) | 88 (16) | 78 (31) | 96 (3.9) | 0.11 | 91 (14) | 90 (15) | 89 (16) | 96 (6.4) | 0.35 |
| Role limitations due to physical health | 77 (35) | 77 (33) | 64 (49) | 89 (21) | 0.30 | 77 (32) | 66 (40) | 88 (21) | 82 (25) | 0.23 |
| Role limitations due to emotional problems | 77 (36) | 87 (22) | 56 (47) | 82 (35) | 0.10 | 74 (37) | 74 (42) | 77 (35) | 73 (36) | 0.97 |
| Energy/fatigue | 55 (19) | 58 (18) | 48 (15) | 57 (23) | 0.43 | 58 (16) | 60 (18) | 57 (12) | 57 (19) | 0.87 |
| Emotional well-being | 71 (17) | 74 (16) | 59 (14) | 77 (16) | 0.043 | 71 (16) | 73 (17) | 69 (16) | 70 (15) | 0.84 |
| Social functioning | 80 (21) | 79 (24) | 72 (23) | 86 (16) | 0.35 | 79 (23) | 72 (31) | 83 (15) | 85 (17) | 0.35 |
| Pain | 82 (21) | 83 (22) | 77 (20) | 84 (21) | 0.73 | 87 (18) | 86 (20) | 81 (15) | 94 (17) | 0.24 |
| General health | 62 (21) | 72 (17) | 51 (17) | 59 (23) | 0.047 | 65 (20) | 69 (22) | 59 (14) | 65 (22) | 0.49 |
| Eq-5d-5l total score | 0.89 (0.13) | 0.90 (0.08) | 0.84 (0.21) | 0.92 (0.07) | 0.37 | 0.93 (0.07) | 0.91 (0.09) | 0.94 (0.05) | 0.93 (0.06) | 0.53 |
| Mobility | 1.4 (0.96) | 1.4 (0.94) | 1.8 (1.3) | 1.1 (0.32) | 0.27 | 1.2 (0.53) | 1.4 (0.74) | 1.1 (0.32) | 1.1 (0.30) | 0.37 |
| Self-care | 1.1 (0.44) | 1.1 (0.36) | 1.2 (0.63) | 1.1 (0.32) | 0.88 | 1.1 (0.24) | 1.1 (0.27) | 1.0 (0.00) | 1.1 (0.30) | 0.66 |
| Usual activities | 1.5 (0.75) | 1.6 (0.76) | 1.6 (0.97) | 1.2 (0.42) | 0.41 | 1.3 (0.54) | 1.4 (0.65) | 1.3 (0.48) | 1.3 (0.47) | 0.75 |
| Pain/discomfort | 1.8 (0.81) | 1.8 (0.80) | 2.0 (0.94) | 1.6 (0.70) | 0.56 | 1.7 (0.64) | 1.8 (0.80) | 1.6 (0.52) | 1.5 (0.52) | 0.63 |
| Anxiety/depression | 1.6 (0.86) | 1.5 (0.65) | 1.8 (1.0) | 1.5 (0.97) | 0.66 | 1.5 (0.70) | 1.6 (0.74) | 1.4 (0.52) | 1.5 (0.82) | 0.72 |
| EQ-5D-5L VAS score | 80 (14) | 81 (12) | 72 (18) | 86 (8.9) | 0.07 | 80 (12) | 79 (14) | 82 (10) | 80 (9.5) | 0.83 |
| Data are in n (%) for categorical data and in mean (sd) for continuous data. ACE III= Addenbrooke’s cognitive examination III. ESS= Epworth Sleepiness Scale. FSS=fatigue severity scale. ISI=insomnia severity index. BDI II= Beck depression Inventory II. mRS=modified ranking scale. Sf-36= short-form-36. EQ-5D-5L= EuroQoL 5-Dimension 5-Level. | | | | | | | | | | |

| Table S5 | | |  | 8 months | | | |  | 20 months | | | |
| --- | --- | --- | --- | --- | --- | --- | --- | --- | --- | --- | --- | --- |
|  |  |  | **Total**  **(n = 39)** | **Mild**  **(n = 14)** | **Moderate**  **(n = 14)** | **Severe**  **(n = 11)** | **P-value** | **Total**  **(n = 39)** | **Mild**  **(n = 14)** | **Moderate**  **(n = 14)** | **Severe**  **(n = 11)** | **P-value** |
| ACE III total score | | | 92 (9.3) | 96 (4.0) | 93 (4.8) | 88 (14) | 0.16 |  |  |  |  |  |
| Attention | | | 18 (0.71) | 18 (0.32) | 18 (0.97) | 18 (0.70) | 0.44 |  |  |  |  |  |
| Memory | | | 24 (3.9) | 25 (1.1) | 24 (2.1) | 22 (6.2) | 0.15 |  |  |  |  |  |
| Fluency | | | 11 (2.9) | 12 (2.2) | 11 (1.9) | 10 (4.0) | 0.35 |  |  |  |  |  |
| Language | | | 25 (2.3) | 26 (0.67) | 26 (0.42) | 24 (3.8) | 0.22 |  |  |  |  |  |
| Visual | | | 15 (1.2) | 15 (1.3) | 15 (1.1) | 14 (1.3) | 0.46 |  |  |  |  |  |
| ESS | | | 6.5 (3.7) | 5.7 (3.4) | 7.1 (5.0) | 7.0 (2.9) | 0.60 | 5.8 (3.7) | 5.2 (3.4) | 7.3 (4.3) | 4.8 (3.1) | 0.22 |
| FSS | | | 3.0 (1.5) | 2.5 (0.91) | 3.1 (1.6) | 3.7 (1.9) | 0.16 | 2.9 (1.3) | 2.3 (1.0) | 3.2 (1.1) | 3.2 (1.7) | 0.14 |
| ISI | | | 7.1 (5.1) | 4.5 (2.9) | 9.0 (5.2) | 8.8 (6.2) | 0.05 | 8.1 (5.8) | 7.5 (5.6) | 9.1 (6.4) | 7.6 (5.7) | 0.77 |
| BDI II | | | 9.2 (7.9) | 7.5 (7.5) | 10 (8.2) | 11 (8.5) | 0.56 | 7.9 (6.1) | 6.1 (5.7) | 9.4 (6.4) | 8.3 (6.1) | 0.39 |
| mRS (mean) | | | 1.1 (0.81) | 0.71 (0.47) | 1.2 (0.89) | 1.4 (0.92) | 0.10 | 0.82 (0.64) | 0.64 (0.50) | 1.0 (0.78) | 0.82 (0.60) | 0.35 |
| mrRS (median) | | | 1.0 [1.0, 2.0] | 1.0 [0.00, 1.0] | 1.0 [1.0, 2.0] | 1.0 [1.0, 2.0] | 0.11 | 1.0 [0.00, 1.0] | 1.0 [0.00, 1.0] | 1.0 [1.0, 1.0] | 1.0 [0.00, 1.0] | 0.45 |
| Barthel index (mean) | | | 100 (1.7) | 100 (0.00) | 99 (2.7) | 100 (0.00) | 0.49 | 100 (2.4) | 100 (0.00) | 99 (4.2) | 100 (0.00) | 0.39 |
| Barthel index (median) | | | 100 [100, 100] | 100 [100, 100] | 100 [100, 100] | 100 [100, 100] | 0.47 | 100 [100, 100] | 100 [100, 100] | 100 [100, 100] | 100 [100, 100] | 0.38 |
| Sf-36 total score | | | 74 (16) | 81 (11) | 77 (10) | 64 (22) | 0.033 | 76 (14) | 82 (11) | 78 (12) | 68 (17) | 0.05 |
| Physical functioning | | | 88 (20) | 91 (13) | 95 (7.2) | 78 (31) | 0.14 | 91 (14) | 93 (14) | 97 (6.2) | 83 (16) | 0.045 |
| Role limitations due to physical health | | | 77 (35) | 94 (15) | 70 (35) | 63 (46) | 0.07 | 77 (32) | 92 (19) | 73 (34) | 63 (36) | 0.07 |
| Role limitations due to emotional problems | | | 77 (36) | 87 (32) | 83 (18) | 57 (47) | 0.10 | 74 (37) | 85 (29) | 81 (33) | 53 (45) | 0.10 |
| Energy/fatigue | | | 55 (19) | 64 (13) | 50 (19) | 48 (22) | 0.08 | 58 (16) | 65 (16) | 55 (15) | 54 (18) | 0.19 |
| Emotional well-being | | | 71 (17) | 75 (14) | 73 (18) | 63 (18) | 0.19 | 71 (16) | 74 (14) | 69 (15) | 68 (20) | 0.61 |
| Social functioning | | | 80 (21) | 86 (18) | 76 (22) | 75 (26) | 0.44 | 79 (23) | 83 (21) | 77 (29) | 78 (20) | 0.81 |
| Pain | | | 82 (21) | 82 (23) | 85 (17) | 78 (22) | 0.79 | 87 (18) | 85 (20) | 89 (17) | 87 (18) | 0.84 |
| General health | | | 62 (21) | 67 (19) | 70 (11) | 49 (25) | 0.034 | 65 (20) | 76 (18) | 66 (15) | 50 (19) | 0.005 |
| Eq-5d-5l total score | | | 0.89 (0.13) | 0.91 (0.09) | 0.90 (0.08) | 0.84 (0.20) | 0.40 | 0.93 (0.07) | 0.95 (0.06) | 0.92 (0.05) | 0.90 (0.10) | 0.21 |
| Mobility | | | 1.4 (0.96) | 1.2 (0.60) | 1.3 (0.90) | 1.9 (1.3) | 0.20 | 1.2 (0.53) | 1.1 (0.28) | 1.0 (0.00) | 1.6 (0.84) | 0.013 |
| Self-care | | | 1.1 (0.44) | 1.1 (0.28) | 1.1 (0.30) | 1.3 (0.67) | 0.43 | 1.1 (0.24) | 1.0 (0.00) | 1.0 (0.00) | 1.2 (0.42) | 0.07 |
| Usual activities | | | 1.5 (0.75) | 1.1 (0.28) | 1.6 (0.50) | 1.8 (1.1) | 0.043 | 1.3 (0.54) | 1.2 (0.44) | 1.3 (0.45) | 1.6 (0.70) | 0.21 |
| Pain/discomfort | | | 1.8 (0.81) | 1.8 (0.83) | 1.7 (0.79) | 1.9 (0.88) | 0.88 | 1.7 (0.64) | 1.4 (0.51) | 1.8 (0.75) | 1.9 (0.57) | 0.13 |
| Anxiety/depression | | | 1.6 (0.86) | 1.6 (0.96) | 1.5 (0.69) | 1.7 (0.95) | 0.81 | 1.5 (0.70) | 1.5 (0.78) | 1.8 (0.62) | 1.3 (0.67) | 0.33 |
| EQ-5D-5L VAS score | | | 80 (14) | 86 (11) | 80 (8.2) | 71 (18) | 0.032 | 80 (12) | 85 (10) | 80 (5.8) | 73 (15) | 0.034 |
|  |  | Data are in n (%) for categorical data and in mean (sd) for continuous data. ACE III= Addenbrooke’s cognitive examination III. ESS= Epworth Sleepiness Scale. FSS=fatigue severity scale. ISI=insomnia severity index. BDI II= Beck depression Inventory II. mRS=modified ranking scale. Sf-36= short-form-36. EQ-5D-5L= EuroQoL 5-Dimension 5-Level. | | | | | | | | | | |

| Table S6  mRS as outcome | 8m,  Or (95-ci) | P-value | 20m,  Or (95-ci) | P-value |
| --- | --- | --- | --- | --- |
| Age | 1.00 (0.96-1.05) | 0.889 | 1.01 (0.97-1.05) | 0.571 |
| Sex | 0.64 (0.14-3.03) | 0.577 | 1.10 (0.28-4.37) | 0.888 |
| Cause: TBEV vs unknown | 0.69 (0.08-5.86) | 0.736 | 0.33 (0.05-2.27) | 0.262 |
| Severity | 1.30 (0.50-3.37) | 0.594 | 1.24 (0.52-2.95) | 0.630 |
| CRP | 1.00 (0.97-1.02) | 0.917 | 1.01 (0.98-1.04) | 0.531 |
| Cell count /m | 1.00 (0.99-1.00) | 0.143 | 1.00 (1.00-1.00) | 0.276 |
| Pathological mri | 1.79 (0.35-9.02) | 0.483 | 1.22 (0.25-5.98) | 0.804 |
| Reduced vigilance | 0.91 (0.19-4.39) | 0.906 | 0.95 (0.22-3.99) | 0.941 |
| Epileptic seizure | 0.50 (0.08-3.31) | 0.472 | 0.36 (0.06-2.12) | 0.259 |
| Cognitive impairment | 3.75 (0.80-17.48) | 0.092 | 1.79 (0.44-7.32) | 0.420 |
| Cranial nerve disorder | 0.81 (0.13-4.91) | 0.817 | 1.09 (0.18-6.58) | 0.928 |
| Vision disorder | 0.50 (0.08-3.31) | 0.472 | 1.32 (0.12-14.14) | 0.819 |
| Language disorder | 1.56 (0.33-7.34) | 0.577 | 1.73 (0.42-7.11) | 0.445 |
| Sensory disorder | 0.98 (0.17-5.82) | 0.982 | 0.82 (0.17-4.00) | 0.804 |
| Paresis | 4.19 (0.46-37.94) | 0.202 | 2.78 (0.51-15.26) | 0.240 |
| Psychiatric disorder | 2.67 (0.29-24.74) | 0.388 | 1.36 (0.23-7.98) | 0.731 |
| Disturbed sleep-wake behaviour | NA |  | 0.36 (0.07-1.98) | 0.240 |
| Logistic model with mrs dichotomized as outcome. The or represent the odds of mrs>0 of the presence of the condition or the increase of a unit (continuous variable). | | | | |

| Table S7  Limitations as outcome | Social 8m,  Or (95-ci) | P-value | Social 20m,  Or (95-ci) | P-value | Prof. 8m,  Or (95-ci) | P-value | Prof. 20m,  Or (95-ci) | P-value |
| --- | --- | --- | --- | --- | --- | --- | --- | --- |
| Age | 1.00 (0.97-1.03) | 0.979 | 0.99 (0.95-1.03) | 0.618 | 0.94 (0.89-1.00) | 0.044 | 1.00 (0.97-1.04) | 0.806 |
| Sex | 1.22 (0.36-4.20) | 0.750 | 4.89 (0.94-25.46) | 0.059 | 0.42 (0.10-1.82) | 0.244 | 1.90 (0.43-8.39) | 0.394 |
| Cause: TBE vs unknown | 0.93 (0.21-4.11) | 0.919 | 0.88 (0.18-4.23) | 0.873 | 1.91 (0.27-13.50) | 0.517 | 0.88 (0.18-4.23) | 0.873 |
| Severity | 2.05 (0.90-4.66) | 0.085 | 2.15 (0.89-5.22) | 0.089 | 1.20 (0.46-3.08) | 0.712 | 1.48 (0.61-3.60) | 0.388 |
| CRP | 0.98 (0.95-1.01) | 0.181 | 1.00 (0.98-1.02) | 0.872 | 1.01 (0.99-1.03) | 0.445 | 1.01 (0.99-1.03) | 0.294 |
| Cell count /m | 1.00 (1.00-1.00) | 0.397 | 1.00 (1.00-1.00) | 0.758 | 1.00 (1.00-1.00) | 0.568 | 1.00 (1.00-1.00) | 0.988 |
| Pathological mri | 1.70 (0.38-7.50) | 0.486 | 1.03 (0.23-4.66) | 0.974 | 2.86 (0.32-25.80) | 0.350 | 0.76 (0.16-3.56) | 0.730 |
| Reduced vigilance | 1.27 (0.36-4.51) | 0.709 | 2.38 (0.63-9.00) | 0.201 | 0.55 (0.10-3.03) | 0.492 | 2.17 (0.54-8.72) | 0.277 |
| Epileptic seizure | 2.77 (0.54-14.23) | 0.223 | 1.05 (0.18-6.26) | 0.953 | 1.89 (0.30-11.77) | 0.497 | 1.38 (0.23-8.33) | 0.727 |
| Cognitive impairment | 3.11 (0.73-13.20) | 0.124 | 1.82 (0.42-7.90) | 0.425 | 0.92 (0.20-4.33) | 0.919 | 6.36 (0.73-55.30) | 0.093 |
| Cranial nerve disorder | 3.75 (0.77-18.29) | 0.102 | 0.85 (0.15-4.86) | 0.854 | 3.30 (0.62-17.62) | 0.162 | 0.41 (0.05-3.80) | 0.436 |
| Vision disorder | 1.07 (0.22-5.17) | 0.932 | 0.85 (0.15-4.86) | 0.854 | 6.80 (1.28-36.26) | 0.025 | 2.33 (0.46-11.85) | 0.310 |
| Language disorder | 1.33 (0.40-4.43) | 0.639 | 2.93 (0.78-10.98) | 0.110 | 0.32 (0.06-1.73) | 0.185 | 3.10 (0.76-12.61) | 0.115 |
| Sensory disorder | 1.67 (0.42-6.59) | 0.466 | 0.98 (0.21-4.43) | 0.974 | 1.88 (0.38-9.20) | 0.438 | 0.67 (0.12-3.68) | 0.642 |
| Paresis | 2.80 (0.75-10.45) | 0.125 | 3.31 (0.84-13.03) | 0.087 | 1.40 (0.29-6.68) | 0.673 | 1.71 (0.41-7.25) | 0.464 |
| Psychiatric disorder | 1.23 (0.29-5.16) | 0.777 | 1.16 (0.25-5.37) | 0.852 | 0.40 (0.04-3.67) | 0.420 | 1.55 (0.33-7.41) | 0.581 |
| Disturbed sleep-wake behaviour | 1.06 (0.31-3.67) | 0.925 | 0.87 (0.23-3.27) | 0.840 | 1.17 (0.25-5.41) | 0.844 | 1.70 (0.38-7.50) | 0.486 |
| Logistic model with social/professional limitations dichotomized as outcome. The OR represent the odds of social limitations of the presence of the condition or the increase of a unit (continuous variable). | | | | | | | | |

| Table S8 | OR (95-CI) | P-value |
| --- | --- | --- |
| Free of complaints | 1.02 (0.97-1.08) | 0.371 |
| Headache (< 15d/month) | 0.96 (0.90-1.02) | 0.213 |
| Cognitive impairment | 0.98 (0.94-1.03) | 0.509 |
| Cranial nerve disorder | 0.92 (0.73-1.14) | 0.437 |
| Aphasia | 1.04 (0.96-1.13) | 0.323 |
| Epileptic seizures | 0.77 (0.37-1.63) | 0.500 |
| Focal motoric deficits | 1.02 (0.94-1.11) | 0.630 |
| Focal sensory deficits | 1.06 (0.95-1.19) | 0.270 |
| Gait disorder | 0.95 (0.84-1.07) | 0.375 |
| Micturition disorder/urinary retention | 0.94 (0.71-1.24) | 0.645 |
| Psychiatric symptoms/diagnoses | 1.00 (0.91-1.10) | 0.954 |
| Excessive fatigue/daytime sleepiness | 0.94 (0.89-1.00) | 0.049 |
| Sleep disorder | 0.93 (0.89-0.98) | 0.009 |
| As efficient/productive as before the disease | 0.99 (0.95-1.04) | 0.824 |
| More quickly exhausted | 1.04 (0.98-1.11) | 0.204 |
| Limited in social life | 0.97 (0.93-1.02) | 0.189 |
| Limited in professional life | 0.96 (0.91-1.01) | 0.105 |
| Logistic model with social/professional limitations dichotomized as outcome. The OR represents the odds when increasing/decreasing the follow-up by one month. | | |
